# G Protein γ subunit 7 loss contributes to progression of clear cell renal cell carcinoma

**DOI:** 10.1002/jcp.28597

**Published:** 2019-04-03

**Authors:** Shan Xu, Haibao Zhang, Tianjie Liu, Yule Chen, Dalin He, Lei Li

**Affiliations:** ^1^ Department of Urology The First Affiliated Hospital of Xi’an Jiaotong University Xi’an P.R. China; ^2^ Oncology Research Lab Key Laboratory of Environment and Genes Related to Diseases Ministry of Education Xi’an P.R. China

**Keywords:** cell cycle, GEO, GNG7, RCC

## Abstract

Clear cell renal cell carcinoma (ccRCC) is a common urinary neoplasm, looking for useful candidates to establish scientific foundation for the therapy of ccRCC is urgent. We downloaded genomic profiles of GSE781, GSE6244, GSE53757, and GSE66271 from the Gene Expression Omnibus (GEO) database. GEO2R was used to analyze the derivative genes, while hub genes were screened by protein‐protein interactions and cytoscape. Further, overall survival, gene methylation, gene mutation, and gene expression were all analyzed using bioinformatics tools. Colony formation and cell‐cycle assay were used to detect the biological function of GNG7 in vitro. We found that *GNG7* was downregulated in ccRCC tissues and negatively associated with overall survival in ccRCC patients. We also found that promoter methylation and frequent gene mutation were responsible for *GNG7* gene suppression. GNG7 low expression was related to upregulation of enhancer of zeste homolog 2 and downregulation of disabled homolog 2‐interacting protein. Further, Gene Set Enrichment Analysis results showed that mTOR1, E2F, G2M, and MYC pathways were all significantly altered in response to GNG7 low expression. In vitro, A498 and 786‐O cells in which GNG7 expression was silenced, exhibited a lower G1 phase when compared to the negative control cells. Taken together, our findings suggest that *GNG7* is a tumor suppressor gene in ccRCC progression and represents a novel candidate for ccRCC treatment.

## 1 INTRODUCTION

Renal cell carcinoma (RCC) is one of the 10 most common cancers in both men and women and clear cell renal cell carcinoma (ccRCC), a type of kidney cancer, accounts for about 85–90% cases (Cho et al., 2016). According to the American Cancer Society, about 63,340 new cases of kidney cancer are estimated to occur in 2018, with the estimated 14,970 deaths in the United States. Although, the von Hippel–Lindau‐hypoxia inducible factor (VHL‐HIF) signaling pathway has been reported as the main cause of ccRCC progression (Frew & Moch, 2015; Ricketts et al., 2016), Young et al. (2009) found no relationship between VHL mutations/deletions and prognosis in ccRCC patients. In addition, several genes such as Polybromo 1, SET Domain Containing 2, and Ras‐related protein 1 were found to be associated with most ccRCC and were found to be responsible for changing the phenotype of normal kidney cells into RCC cells (W. Gao, Li, Xiao, Liu, & Kaelin, 2017; Gossage et al., 2014; Murakami et al., 2017).

With the development of bioinformatics, hub genes, relationship between genes, protein‐protein interactions (PPIs), and functional pathways involved in the cancer carcinogenesis have provided valuable information about the pathogenesis of ccRCC. In 2003, Lenburg et al. (2003) compared seven cohorts of ccRCC and observed differential expression in 24.8–82.9% of genes. In 2007, Gumz et al. (2007) reported that secreted frizzled‐related protein 1 is a tumor suppressor by analyzing genomic profiling of ccRCC tumors and patient‐matched normal tissues. In 2014, neuronal pentraxin 2 was found to be overexpressed, specifically in ccRCC (von Roemeling et al., 2014). In 2016, Zofia et al. constructed a network of microRNA (miRNA) and messenger RNA (mRNA) involved in ccRCC progression (Wotschofsky et al., 2016). However, the core genes identified in different cohorts were different (Gumz et al., 2007; Lenburg et al., 2003; von Roemeling et al., 2014; Wotschofsky et al., 2016). Thus, further studies are needed to comprehensively and systematically identify possible prognostic, diagnostic, and therapeutic targets of differential gene expression in three or more cohorts. In our study, the differentially expressed genes (DEGs) of GSE781, GSE6244, GSE53757, and GSE66271 were obtained and the hub genes were screened on the basis of different clinical databases, PPI, and cytoscape. We found that G Protein γ subunit 7 (*GNG7*) plays a key role in ccRCC progression and is associated with the mTOR pathway. Thus, *GNG7* gene may be a potential target for detection of an early onset of ccRCC and may serve as target for the treatment of ccRCC.

## 2 MATERIALS AND METHODS

### 2.1 Microarray data

GSE781, GSE6244, GSE53757, and GSE66271 were applied from the Gene Expression Omnibus (GEO), nine ccRCC patients‐tumor tissue and matched normal tissue were obtained after surgery in GSE781 data set on a GLP96/97 platform (Lenburg et al., 2003); GSE6344 included 10 patient‐matched normal renal cortex and ccRCC tissues on a GLP96/97 platform (Gumz et al., 2007); GSE53757 was performed by von Roemeling et al. (2014) and 72 ccRCC patient‐tumor tissue and matched normal tissue were collected. Gene expression was compared between tumor and matched normal samples on a GPL570 platform; GSE66271 was composed of 13 ccRCC patient‐tumor tissue and matched normal tissue on a GPL570 platform (Wotschofsky et al., 2016). DEGs between tumor and matched normal samples were analyzed by GEO2R, an online analysis tool in the GEO website.

### 2.2 Venn analysis

Log (fold‐change) > 1 and adj. *p* ≤ 0.01 DEGs in four cohorts were considered statistically significant, and submitted to Bioinformatics & Systems Biology to obtain the statistically significant DEGs. Bioinformatics & Systems Biology is a web in the fields of gene prediction and genome annotation, comparative and evolutionary genomics, and systems biology.

### 2.3 Hub gene analysis

PPI was constructed by the Search Tool for the Retrieval of Interacting Genes (STRING; http://string.embl.de/) to analyze the interaction of DEGs with the medium confidence at 0.45. Then, cytoscape (version 3.4.0), an open source software platform for data integration, analysis, and visualization, was used to analysis DEGs (Shannon et al., 2003). The overall survival and gene expression in different stage/grade renal cell carcinoma were performed in UALCAN (http://ualcan.path.uab.edu). UALCAN is an interactive web resource for analyzing and generating graphs with The Cancer Genome Atlas (TCGA) transcriptome data using javascript and CSS (Chandrashekar et al., 2017). Methylation analysis was performed in the human pan‐cancer methylation database (MethHC, http://methhc.mbc.nctu.edu.tw/php/index.php). MethHC is a web resource, focused on the DNA methylation and gene expression from TCGA (Huang et al., 2015). Gene mutation was performed in cBioPortal (http://www.cbioportal.org). cBioPortal, a user‐friendly web resource for Cancer Genomics, provides visualization, analysis, and download of large‐scale cancer genomics data sets (Cerami et al., 2012; J. J. Gao et al., 2013).

### 2.4 Gene Set Enrichment Analysis

The RNA‐seq data of 611 ccRCC patients were download from the TCGA database (September 10, 2018) and GNG7 high‐expression group (top 135 [25% of 611] high‐YAP expression patients) and GNG7 low‐expression group (top 134 [25% of 611] low‐GNG7 expression patients) were set up. Then, mRNA expression data of the two groups were submitted to Gene Set Enrichment Analysis (GSEA) 2.0 software (Subramanian et al., 2005), and the hallmark gene sets (http://software.broadinstitute.org/gsea/msigdb/collections.jsp#H) were selected for analysis. Hallmark gene sets summarize and represent specific well‐defined biological states or processes and display coherent expression including 50 hallmarks, which condense information from over 4,000 original overlapping gene sets from v4.0 MSigDB collections C1 through C6 (Liberzon et al., 2015; Liberzon et al., 2011).

### 2.5 Cancer Cell Line Encyclopedia analysis

Cancer Cell Line Encyclopedia (CCLE; https://portals.broadinstitute.org/ccle/home) database, is a publicly accessible online microarray database (Barretina et al., 2012). *GNG7* was submitted to CCLE to analyze GNG7 mRNA expression in different cell lines and different cancer types.

### 2.6 Cell lines and cell culture

Human renal cancer cell lines, A498 and 786‐O, were purchased from the American Type Culture Collection (Manassas, VA), and maintained in RPMI 1640 medium (Gibco; Thermo Fisher Scientific, Inc., Waltham, MA) with 10% (v/v) fetal bovine serum (Gibco; Thermo Fisher Scientific, Inc., Waltham, MA) at 37°C in a humidified 5% CO_2_ incubator.

### 2.7 Oligo small interfering RNA transfection

Cells were cultured in six‐well dishes for 24 hr, then transfected with oligo small interfering RNA (siRNA) against GNG7 (RiboBio, Guangzhou, China) using Roche X‐tremeGENE siRNA transfection reagent (Roche Co. Ltd., Shanghai, China). The knockdown was verified by western blot analysis and real‐time quantitative polymerase chain reaction.

### 2.8 Real‐time quantitative polymerase chain reaction

Total RNA isolation and cDNA synthesis was carried out using the RNA fast 200 kit (Feijie Biotech, Shanghai, China) and Prime Script™ RT reagent kit (Takara Biotechnology Co. Ltd., Dalian, China), respectively. SYBR Green PCR Master Mix (Takara Biotechnology Co. Ltd., Dalian, China) was used to detect relative gene expression, calculated by the $2^{-\Delta \Delta C_{t}}$ method using glyceraldehyde 3‐phosphate dehydrogenase (GAPDH) as a reference gene (Livak & Schmittgen, 2001). GAPDH and GNG7 primers sequences were the following: GAPDH forward, 5′‐ATGGGGAAGGTGAAGGTCGG‐3′, reverse, 5′‐GACGGTGCCATGGAATTTGC‐3′; GNG7 forward, 5′‐CGTCTGACCTCATGAGCTACTGTGA‐3′, reverse, 5′‐CAAGGTTTCTTGTCCTTAAAGGGGTTC‐3′.

### 2.9 Western blot analysis

The western blot analysis protocol was performed as described previously (Xu et al., 2013). Antibody against GNG7 was purchased from ABclonal (#A10009, 1:1000; ABclonal, Wuhan, China), and anti‐β‐actin antibody (#JB09, 1:1,000; Absin, Shanghai, China) was used for detection of actin as an internal control. After 24 hr, incubated horseradish peroxidase‐conjugated secondary antibodies (peroxidase‐conjugated affiniPure goat anti‐rabbit IgG (#ZB‐2301, 1:2,000; Beijing Zhongshan Golden Bridge Biotechnology, Co. Ltd., Beijing, China) and peroxidase‐conjugated affiniPure goat anti‐mouse IgG (#ZB‐2305, 1:2,000; Beijing Zhongshan Golden Bridge Biotechnology, Co. Ltd., Beijing, China) for 1 hr at room temperature. Immunoreactive signals were detected by a Western Bright Quantum HRP substrate kit (Advansta, Inc., Menlo Park, CA), visualized by a Molecular Imager ChemiDoc XRS system (Bio‐Rad Laboratories, Inc., Hercules, CA).

### 2.10 MTT assay

Cell proliferation in GNG7 knockdown was carried out as previously described (Xu et al., 2016). Cells (4,000 cells per well) were plated in a 96‐well plate for 48 hr and cell viability was assessed using 3‐(4,5‐dimethyl‐2‐thiazolyl)‐2,5‐diphenyl‐2‐H‐tetrazolium bromide (MTT). The growth rate was calculated as the average OD value in GNG7‐silenced cells group)/average OD value in the control group × 100%.

### 2.11 Clone and clonogenic assay

Cells transfected with oligo siRNA were seeded in six‐well plates (1,000 cells/well) and cultured for 5 days. Crystal violet (0.5% m/v) was then used to stain the cells and images were captured by a microscope.

### 2.12 Cell‐cycle analysis

Cell transfected with oligo siRNA A498 and 786‐O were harvested, washed with phosphate‐buffered saline, and fixed overnight in 70% ethanol at 20°C. Cells were incubated with propidium iodide at room temperature for 30 min as previously described (Xu et al., 2013), and were analyzed by flow cytometry using a FACSCalibur flow cytometer (Becton, Dickinson and Company, BD Biosciences, San Jose, CA). The data were analyzed using the cell fit software.

### 2.13 Statistical analysis

The differences between two groups (Student's *t* test) were analyzed by GraphPad Prism version 6.0 software (GraphPad), *p *< 0.05 was considered significant.

## 3 RESULTS

### 3.1 The hub genes in clear cell renal cell carcinoma progression

**Figure 1:**
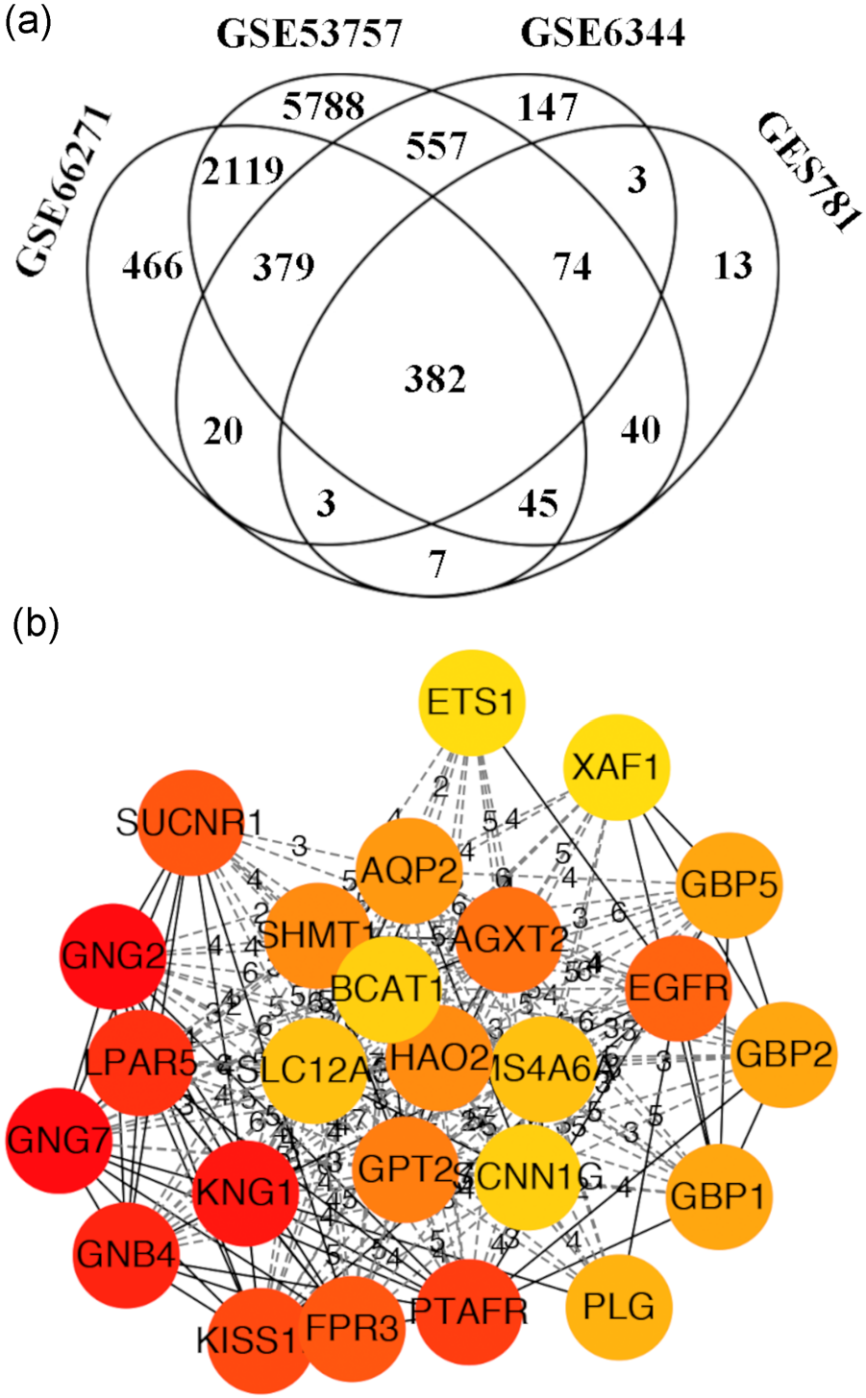
Venn diagram and protein‐protein interaction (PPI) analysis differentially expressed genes (DEGs). (a) DEGs were selected with Log (fold‐change) > 1 and adj. *p* ≤ 0.01 among GSE781, GSE6344, GSE53757, and GSE66271 clear cell renal cell carcinoma (ccRCC) microarray data sets. (b) The coexpression network of DEGs was constructed by PPI and visualized by cytoscape and the significant module was marked in light red [Color figure can be viewed at wileyonlinelibrary.com]

To identify the hub genes involved in ccRCC progression, gene profiling was performed between tumor tissue and the matched tissue and the DEGs were extracted from each data set. We identified 740 genes in GSE781, 2,231 genes in GSE6344, 17,082 genes in GSE53757, and 5,223 genes in GSE66271 that were differentially expressed by Log (fold‐change) > 1 with *p* ≤ 0.01. Furthermore, a four‐way Venn diagram of GSE781, GSE6344, GSE53757, and GSE66271 data sets revealed that 382 explicit genes were commonly identified (Figure 1a, Supporting Information 1).

**Figure 2:**
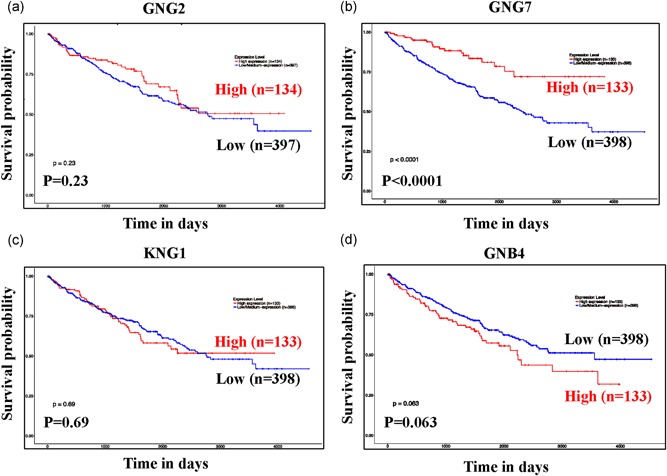
Kaplan‐Meier survival curve for four hub genes associated with ccRCC overall survival. Overall survival of GNG2 (a), GNG7 (b), KNG1 (c), and GNB4 (d) were performed by using UALCAN online platform. *p* < 0.05 was considered statistically significant. ccRCC: clear cell renal cell carcinoma [Color figure can be viewed at wileyonlinelibrary.com]

Next, the interaction among the 382 explicit genes was constructed by the PPI network using STRING (Supporting Information 2) and the significant gene module was calculated by cytoscape (Figure 1b). Results showed that *GNG2*, *GNG7*, *KNG1*, and *GNB4* genes were identified as hub genes with maximal clique centrality (MCC) value > 1,000 and degree ≥ 10 (Table 1). Subsequently, overall survival analysis of hub genes was performed by UALCAN Figure 2a). GNG7 mRNA expression had a significant effect on ccRCC patient overall survival (*p* < 0.0001). In contrast, GNG2, KNG1, and GNB4 mRNA expression showed much less effect on ccRCC patient overall survival (*p* > 0.05). Based on these results, we further analyzed *GNG7* gene in subsequent experiments.

**Table 1:** Four hub genes with MCC value > 1,000 and degree ≥ 10

Nos.	Gene symbol	Full name	Function
1	GNG2	G Protein subunit γ 2	GNG2 inhibits proliferation of malignant melanoma cells in vitro and in vivo
2	GNG7	G Protein subunit γ 7	Low‐expression GNG7 is associated with tumor grade and stage
3	KNG1	Kininogen 1	Kininogen‐1 is a constituent of the blood coagulation system as well as the kinin‐kallikrein system
4	GNB4	G protein subunit β 4	*GNB4* mutations as a cause of Dominant Intermediate Charcot‐Marie‐Tooth Disease

### 3.2 GNG7 expression is repressed in the tumor

**Figure 3:**
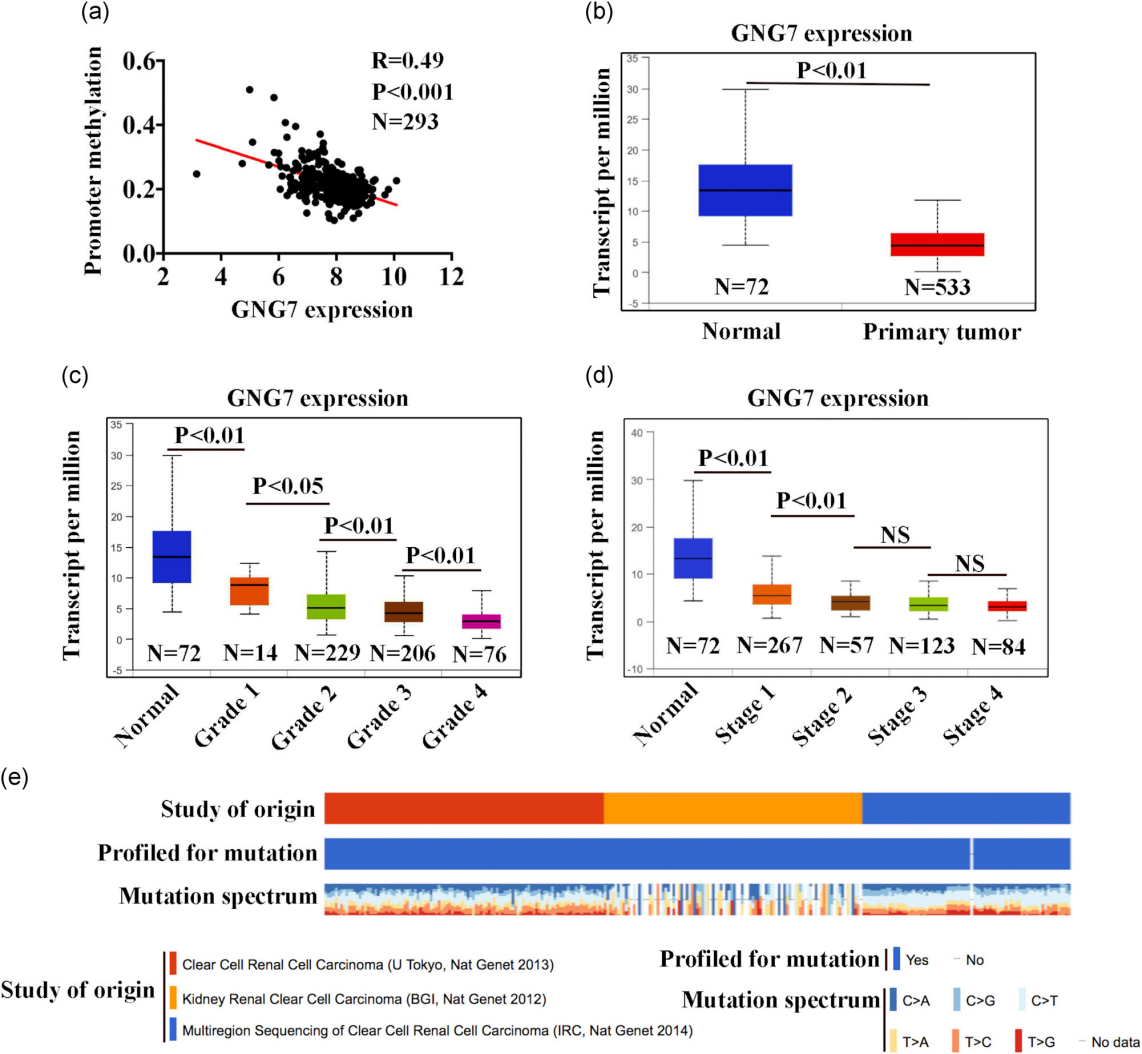
Expression of GNG7 related to clinical features according to patients’ clinicopathological characteristics. (a) The mutation in *GNG7* gene in ccRCC patient genome was analyzed in three cohorts using the cBioPortal online platform. (b) *GNG7* gene promoter methylation information in 293 ccRCC patients was obtained from MethHC web resource by analyzing The Cancer Genome Atlas (TCGA) database. UALCAN online platform analysis of normal versus primary tumor of GNG7 (c), and association between the expression of GNG7 and tumor grade (d), and tumor stage (e) were also performed. *p* < 0.05 was considered statistically significant. ccRCC: clear cell renal cell carcinoma [Color figure can be viewed at wileyonlinelibrary.com]

TCGA ccRCC patient gene array showed that GNG7 mRNA expression was significantly repressed in tumor tissue in comparison to the normal tissue (Figure 3a). Further, we analyzed the expression level of GNG7 in normal and ccRCC tissue samples from grades 1, 2, 3, 4, and stages I, II, III, IV. We found that GNG7 was significantly downregulated in ccRCC in all grades (*p* < 0.05; Figure 3b). However, no significant difference was found in the stages II, III, and IV (Figure 3c), and GNG7 was expressed in higher levels when compared to the normal tissues.

To investigate the mechanism of the low gene expression of *GNG7* in ccRCC tissue, DNA methylation, an important epigenetic regulator of gene transcription, was analyzed by MethHC. We found a high level of DNA methylation in *GNG7* gene promoter region, which silenced *GNG7* gene at the transcriptional level in ccRCC tissues, based on TCGA data set (Figure 3d). Further, DNA mutation analysis was performed using cBioPortal, which showed that nucleotide C was increased while nucleotide T was decreased in ccRCC tissues (Figure 3e).

### 3.3 Pathway enrichment analysis for GNG7 in ccRCC

With the aim of identifying the role of GNG7 in ccRCC progression, RNA‐seq data from 611 ccRCC patients were download from TCGA database (September 10, 2018) and segregated into GNG7 high‐expression group (top 135 [25% of 611] high‐GNG7 expression patients) and GNG7 low‐expression group (top 134 [25% of 611] low‐GNG7 expression patients). The data from the two groups were submitted to GSEA 2.0 software for “hallmark gene sets” (browse 50 gene sets) enrichment analysis.

**Figure 4:**
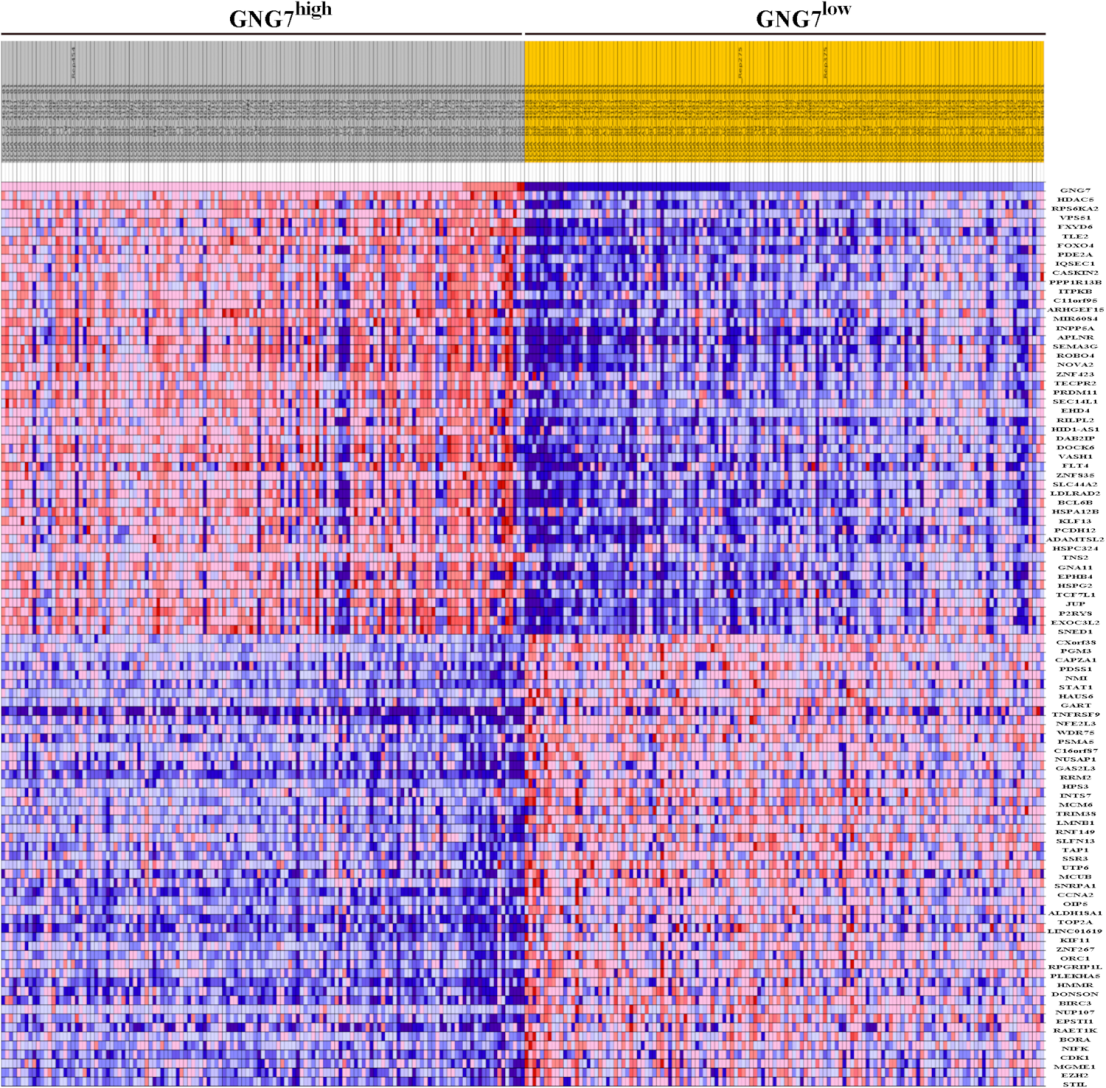
Heat map of top 100 genes induced or repressed in GNG7 high‐expression and GNG7 low‐expression ccRCC patient groups. Patient information was download from TCGA database (September 10, 2018), and separated into GNG7 high‐expression group (25% of 611 patients) and GNG7 low‐expression group (25% of 611 patients). For each group, 50 significantly changed genes were reported and represented by heat map. Range of colors (red, pink, light blue, dark blue) shows the range of expression values (high, moderate, low, lowest, respectively). ccRCC: clear cell renal cell carcinoma; TCGA: The Cancer Genome Atlas [Color figure can be viewed at wileyonlinelibrary.com]

**Figure 5:**
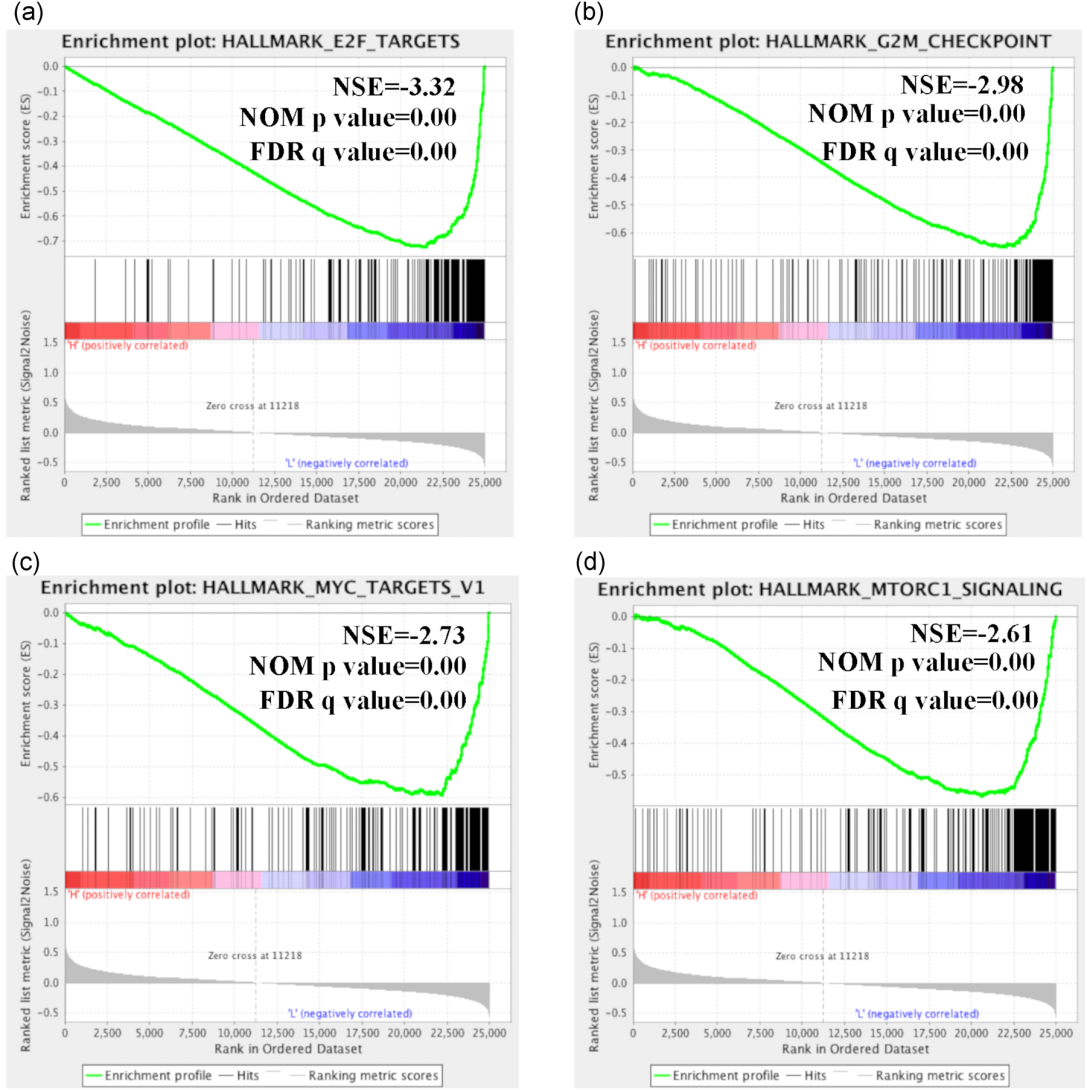
The four Gene Set Enrichment Analysis (GSEA) results of significantly altered cell signaling pathways is response to decreased GNG7 expression. Information of 611 ccRCC patients was download from TCGA database (September 10, 2018), and separated into GNG7 high‐expression group (25% of 611 patients) and GNG7 low‐expression group (25% of 611 patients). The data from the two groups were submitted to GSEA, and the effect on 50 hallmark gene sets was analyzed for each group. (a) *“*hallmark_E2F_targets,” (b) “hallmark_G2M_ckeckpoint,” (c) “hallmark_MYC_targets_v1,” and (d) “hallmark_MTORC1_signaling”. ccRCC: clear cell renal cell carcinoma; TCGA: The Cancer Genome Atlas [Color figure can be viewed at wileyonlinelibrary.com]

The profile of top 50 genes for each phenotype was found to be completely different among the GNG7 high‐expression and GNG7 low‐expression groups (Figure 4). GNG7 low expression was associated with the activation of oncogenes such as enhancer of zeste homolog 2 (EZH2), cyclin‐dependent kinase 1 (CDK1), signal transducer and activator of transcription 1 (STAT1), and transporter associated with antigen processing 1 (TAP1). In contrast, tumor suppressor genes such as disabled homolog 2‐interacting protein and forkhead box protein O4 (FOXO4) were found to be downregulated in the GNG7 low‐expression group. For a better identification of biological processes involved in the whole network of genes, the enrichment plot was used that provides a graphical view of the enrichment score for each gene set. We found that the distribution curves tended to be “bumpy” in phenotype in GNG7 expression high‐group enrichment when compared to the enrichment plot of GNG7 low expression group (Figure S3). Further, 34 of 50 gene sets were upregulated in GNG7 low‐expression group. After a normalized enrichment score, nominal *p*‐value, and false discovery rate *q*‐value analysis, we found the gene sets of “hallmark_E2F_targets,” “hallmark_G2M_ckeckpoint,” “hallmark_MYC_targets_v1,” and “hallmark_MTORC1_signaling” were responsible for the lower expression GNG7 in progress biological behavior of ccRCC (Figure 5). These results reveal that repressed GNG7 was associated with increased cell proliferation and viability in ccRCC progression.

### 3.4 GNG7 dampens proliferation of ccRCC cell lines

**Figure 6:**
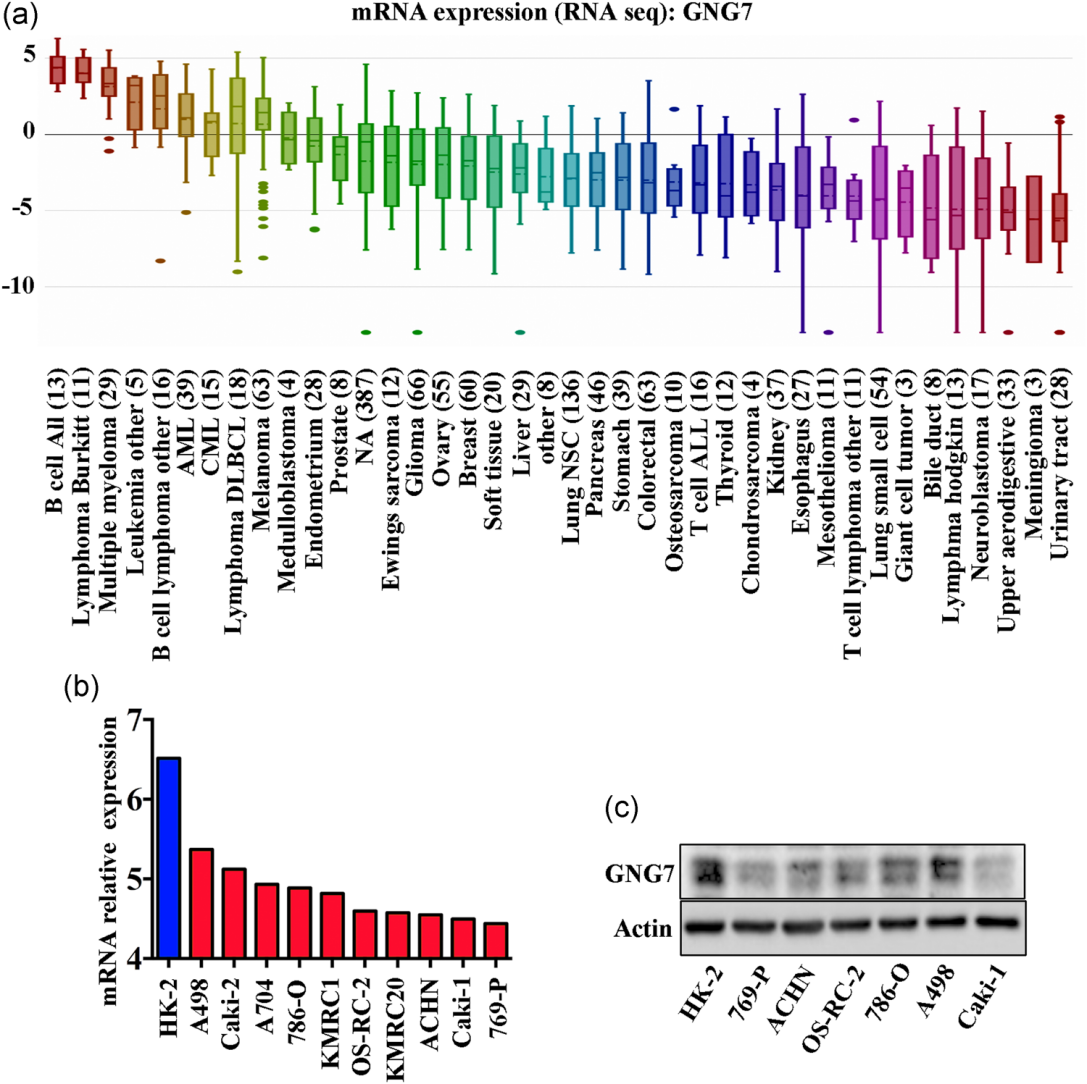
GNG7 is downregulated in ccRCC cell lines. Cancer Cell Line Encyclopedia was used to compare the messenger RNA expression of GNG7 in different tumor cells (a), kidney normal cell HK‐2, and other ccRCC cell lines (b). HK‐2, 769‐P, ACHN, OS‐RC‐2, and 786‐O cell lysates were collected and GNG7 protein expression was analyzed by western blot, β‐actin was used as an internal control. ccRCC: clear cell renal cell carcinoma [Color figure can be viewed at wileyonlinelibrary.com]

**Figure 7:**
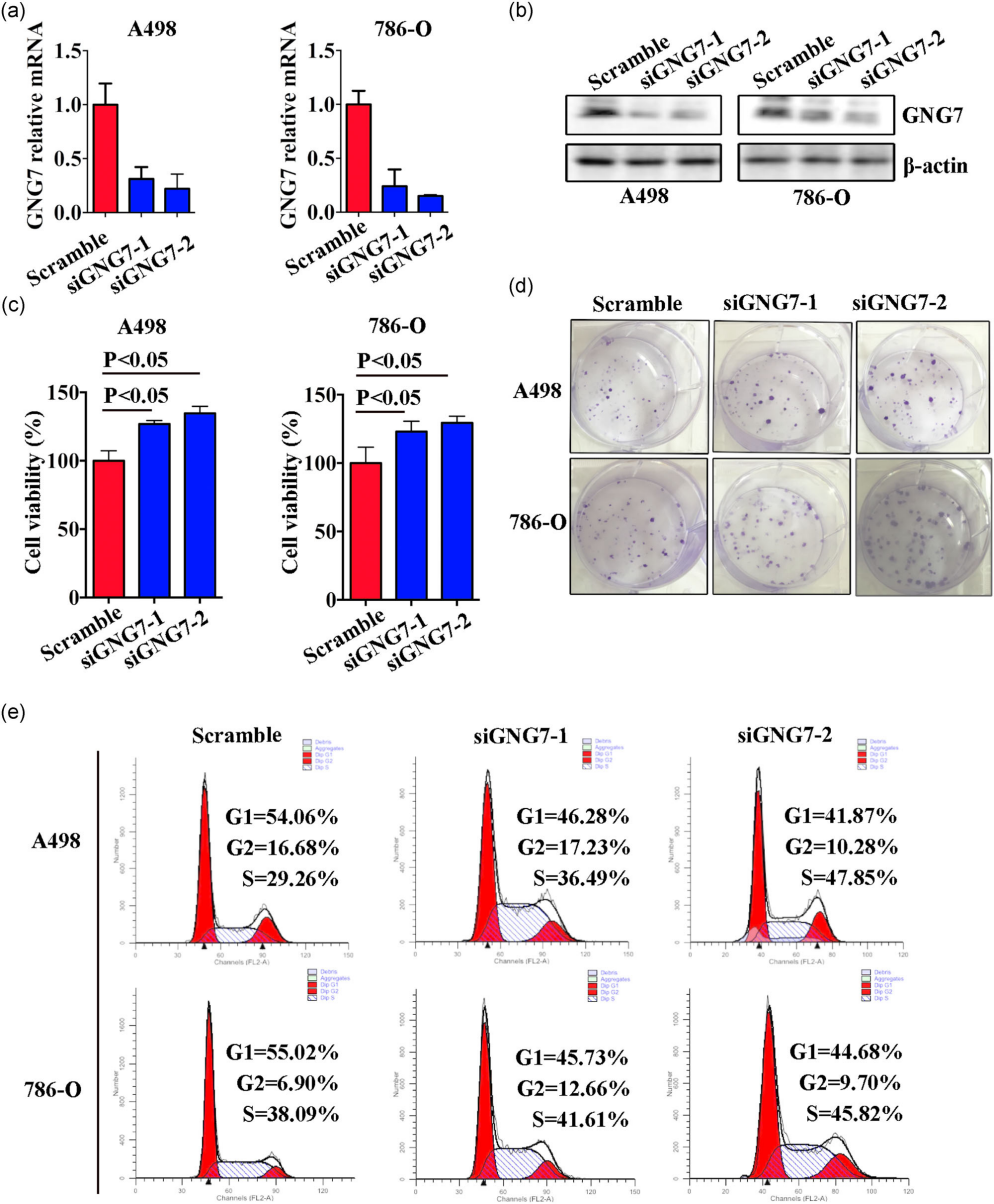
Loss of GNG7 facilitated cell proliferation by increasing G2/M cell‐cycle phase. A498 and 786‐O cells were transfected with oligo siGNG7 or negative control oligo small interfering RNA (siRNA). After 48 hr, cells lysates were collected and expression of GNG7 was analyzed by real‐time quantitative polymerase chain reaction (a) and western blot analysis (b). (c) MTT assay was used to detect cell viability in A498 and 786‐O cells transfected with GNG7 siRNA. (d) The colony formation ability in A498 and 786‐O cells was visualized by crystal violet staining. (e) Flow cytometric analysis was used to detect cell cycle of A498 and 786‐O cells after transfection. *p* < 0.05 was considered statistically significant [Color figure can be viewed at wileyonlinelibrary.com]

To investigate the effect of GNG7 on ccRCC cell proliferation and viability, we used a CCLE database, which demonstrated that the GNG7 mRNA expression level was low in many cancer types (Figure 6a). Meanwhile, the mRNA expression level of GNG7 in proximal tubular cell line (HK‐2) was significantly higher than that in ccRCC cell lines (Figure 6b,c). Next, we performed a series of in vitro experiments using a loss‐of‐function of GNG7 in ccRCC cell lines. We found that the cell growth rate was increased in A498 and 786‐O cells transfected with siRNA against GNG7 when compared to the negative control (Figure 7c). Colony formation assay showed a dramatically increased number of colonies in GNG7‐silenced cells in comparison to the negative control cells (Figure 7d). To determine the mechanism of cell growth inhibition in by GNG7, cell‐cycle assay was performed in A498 and 786‐O cells after knocking down GNG7 for 48 hr (Figure 7e). Cells in G1 phase were 55.02% and 54.06% in negative control in A498 and 786‐O cells, respectively. On the other hand, we found 41.87 and 46.28% of cells in G1 phase in A498 GNG7‐silenced cells (siGNG7‐1 and siGNG7‐2), and 45.73 and 44.68% cells in 786‐O GNG7‐silenced cells (siGNG7‐1 and siGNG7‐2).

## 4 DISCUSSION

In this study, we identified 25,276 genes that were significantly differentially expressed at Log (fold‐change) > 1 and adj. *p* ≤ 0.01 in ccRCC tumor tissue relative to the matched tissue. A total of 382 DEGs were identified among the four cohorts (Figure 1a), cytoscape and PPI network were performed to explore the gene coexpression network among the DEGs. We selected four DEGs (*GNG2*, *GNG7*, *KNG1*, and *GNB4*) as hub genes with MCC value ≥ 1,000 combing degrees ≥10 (Figure 1b). Further validation in TCGA data set showed that low expression of *GNG7* gene was related to a worse overall survival (Figure 2b), but, *GNG2*, *KNG1*, and *GNB4* genes had no significant effect on ccRCC patient overall survival (Figure 2a,c, and d). These results suggest that *GNG7* gene is a tumor suppressor in ccRCC.

*GNG7* belongs to the large G protein γ family (Shibata, Mori, Tanaka, Kitano, & Akiyoshi, 1998). It was reported that *GNG7* is a tumor suppressor gene in esophageal cancer, squamous cell carcinoma of head and neck, pancreatic cancer, and gastrointestinal cancer (Hartmann et al., 2012; Long, Liu, Wu, Xu, & Ge, 2016; Shibata et al., 1998). However, the role of *GNG7* in cancer is poorly understood and the significance of *GNG7* gene expression in ccRCC remains unknown.

Consistent with the previous studies (Long et al., 2016; Ohta et al., 2008; Shibata et al., 1998), we observed that a lower GNG7 expression was significantly associated with an overall poor survival and high grade/stage in ccRCC patients (Figure 2b, 3d, and 3e). Regarding the clinical pathological parameters, we found that low expression of GNG7 was significantly associated with tumor grade. These findings are consistent with the previous reports showing that loss of GNG7 was related to large tumor and tumor invasion and aggressiveness in squamous cell carcinoma of head and neck and esophageal cancer (S. Wu, F. Wu, & Jiang, 2017; Ohta et al., 2008), respectively.

*GNG7* gene has a highly methylated promoter in squamous cell carcinoma of the head and neck and esophageal cancer (Hartmann et al., 2012; Ohta et al., 2008), however, the effect and underlying mechanism of GNG7 loss and the function on cancer biology in ccRCC remain unknown. In this study, we found that *GNG7* gene promoter was highly methylated in tumors but unmethylated in normal tissues as shown by TCGA data set (Figure 3b). Moreover, *GNG7* gene mutation was found in almost all ccRCC patients in this study, further validating the TCGA data set (Figure 3a). This result indicates that GNG7 gene methylation and high CG sit may be responsible for GNG7 gene inactivation in ccRCC progression. The expression of GNG7 may be regulated by miR‐328 and demethylation drug 5‐AZAC restored GNG7 expression in other cancer cell lines (Ohta et al., 2008), but the molecular mechanism of such effects in ccRCC remain unclear and warrant further investigation.

Our GSEA analysis showed that GNG7 has a significant effect on mTOR1 signaling which is the downstream of VHL‐HIFs signaling pathway in ccRCC. Further, the downregulated GNG7 gene mainly affected cell proliferation‐related pathways among which the top three were “hallmark_E2F_targets,” “hallmark_G2M_ckeckpoint,” and “hallmark_MYC_targets_v1”. As GNG7 affected mTOR1 signaling, we speculate that GNG7 is an upstream regulator of mTOR1, which may serve as a novel target for the developing diagnostic and therapeutic strategies in ccRCC. All these results predicted by bioinformatics analysis were validated in ccRCC cell lines, where we found that GNG7‐silenced cells grew faster and the G2M phase was increased when compared to the negative control cells, consistent with GNG7 function in other tumors (Liu et al., 2016).

In addition, loss of GNG7 was found to be associated with upregulation of several key genes including EZH2, CDK1, STAT1, and TAP1. EZH2 is linked to many tumors (Wagener et al., 2010) and is a powerful independent predictor of RCC‐related death (Lee & Choe, 2012; Wagener et al., 2010). CDK1, STAT1, and TAP1 have also been reported as oncogenes in many cancers (Adamkova, Souckova, & Kovarik, 2007; Malumbres & Barbacid, 2009; Qian et al., 2017). Meanwhile, DAP2IP, a tumor suppressor, plays an important role in drug resistant by regulating mTOR (Zhou et al., 2016). These above results indicate that GNG7 may play an important role in RCC progression, metastasis, immune control, and in drug resistance.

Conclusion, our study identified the key genes in ccRCC progression and *GNG7* gene was screened from four ccRCC cohorts. *GNG7* gene was strongly suppressed in ccRCC tumor tissues as a result of promoter methylation and frequent gene mutation. GNG7 expression was negatively correlated with ccRCC patient grade and overall survival. Decreased expression of GNG7 was related to mTOR1, E2F, G2M, and MYC pathway. In addition, lower GNG7 expressing ccRCC cells showed an increase in G2/M cell‐cycle phase. These findings suggest that GNG7 is a tumor suppressor in ccRCC progression and has a potential to be a new biomarker or therapeutic target in ccRCC.

## FUNDING

This study was supported by the National Natural Science Foundation of China (NSFC No. 81602244 to Shan Xu) and Natural Science Basic Research Plan in Shaanxi Province of China (Program No. 2017JM8018 to Shan Xu).

## CONFLICT OF INTERESTS

The authors declare that there is no conflict of interests.

## AUTHOR CONTRIBUTIONS

L. L. conceived and supervised the study. S. X., H. Z., and T. L. performed bioinformatics analysis. Y. C. downloaded the GEO data. S. X. wrote the manuscript. D. H. edited the manuscript.

## Supporting information

Supporting information

Supporting information

Supporting information

Supporting information
