# Supplementary material for: G Protein γ subunit 7 loss contributes to progression of clear cell renal cell carcinoma
Source: J Cell Physiol. 2019 Apr 3;234(11):20002–12. doi: 10.1002/jcp.28597 (PMC6767067; doi:10.1002/jcp.28597)
Supplement: Supplementary file 1 — Supporting information [file JCP-234-20002-s001.pdf]

DEGs list

ABHD14B	GLYCTK	QPRT
ACADSB	GNB4	RALGAPA2
ACSL5	GNG2	RASD1
ACSM2B	GNG7	RASSF3
ADAP2	GPAT3	RCSD1
ADGRF1	GPR155	RDH10
ADGRV1	GPT2	RDH12
AFAP1L1	HACL1	REEP6
AGMAT	HAO2	RELL1
AGPAT3	HEPACAM2	RIF1
AGXT2	HES4	RNASET2
AKNA	HLA-DOA	RNF145
ALDOA	HOGA1	RNF149
ALDOB	HOXB-AS3	RNF150
ALS2CL	HS6ST2	RNF212B
ANGPT2	HSD17B14	RPL22L1
ANGPTL4	HSPB2-C11orf52	RPL35A
ANKRD37	HYKK	SAMD5
AP1S2	IAH1	SAMHD1
APBB1IP	IGSF11	SAT2
AQP11	IRX2	SCN3A
AQP2	IRX3	SCN7A
ARHGAP24	ITGA4	SCNN1G
ARHGAP9	IVD	SCRN2
ARL6IP6	IYD	SDSL
ARRDC2	JAZF1	SEMA5B
ASAP1	KCNE1	SEMA6D
ASPDH	KCNJ10	SETD7
ATP6V1G3	KCNJ15	SFXN2
ATPIF1	KIAA0930	SGK2
BCAP29	KIAA1161	SHMT1
BCAT1	KIF16B	SIPA1L2
BHMT2	KIF9	SIX4
BICD1	KISS1R	SLAMF7
BICDL1	KLHL13	SLC12A3
BIRC3	KLHL6	SLC13A2
BSND	KNG1	SLC13A3
C12orf56	LDHD	SLC15A4
C15orf59	LINC00982	SLC16A10
C16orf89	LINC01187	SLC16A7
C1orf162	LIPH	SLC16A9
C1orf168	LIX1L	SLC22A12
C1orf226	LOC100130691	SLC22A7
C1QC	GLYATL1	SLC22A8
C3orf58	LOC100505985	SLC23A1
CAMK1D	AK4	SLC25A33
CASP2	LOC100509445	SLC26A7

CASZ1	LOC101927933	SLC30A2
CCSAP	LOC101928615	SLC38A1
CD84	LOC101928927	SLC39A5
CDCA2	LOC101929036	SLC41A2
CDCA7L	LOC101930349	SLC43A2
CDKN2B	LOC105375115	SLC47A2
CGN	LOC105377924	SLC4A9
CHDH	LOC149703	SLC51B
CHST14	LOC153684	SLC5A11
CISH	LOC154761	SLC6A19
CLCN5	LOC389332	SLC7A13
CLMN	LOC645321	SLFN11
CMPK2	LOC727944	SLIT2
CMTM4	LPAR5	SMCO3
CNRIP1	LYPLAL1	SMIM24
COL8A1	MAP1B	SMIM5
CPNE2	MAP6	SMTNL2
CRNDE	MARCKS	SNHG1
CTSS	MCOLN3	SNORA16A
CTXN3	MFSD4A	SNX20
CXorf36	MIOX	SOBP
CYP8B1	MIR34A	SORCS1
CYS1	MIR4435-2HG	SOST
CYSTM1	MIR6778	SPATA17
DANCR	MPEG1	SSPN
DCLK1	MPP7	ST3GAL1
DERL1	MRLN	ST8SIA4
DHDH	MRO	STAMBPL1
DHRS4-AS1	MRPS25	STK10
DMRT2	MS4A4A	STK32A
DNAJC12	MS4A6A	STMN3
DNER	MS4A7	SUCNR1
DPP6	MTURN	SULT1C2
DTX3L	MUC15	SUSD3
DUSP15	NAPEPLD	SUSD4
EDIL3	NAPSA	SYNE4
EGFR	NAV1	SYT6
EGLN3	NCK1	TAP2
EHF	NETO2	TBC1D24
ENPP6	NFATC2	TBXAS1
ENTPD1	NKAIN4	TCF21
EPB41L5	NOX4	TCF4
ERGIC1	NR3C1	TFCP2L1
ERICH4	NRK	THRB
ERP27	NTNG1	TINAG
ESPN	NUDT6	TKFC
ESRRB	OSBPL6	TLR8
ESYT2	OSMR	TMCC1

ETS1	P2RX7	TMED7-TICAM2
FAM124A	PAG1	TMEM173
FAM167A	PALM3	TMEM174
FAM169A	PAPPA	TMEM178A
FAM26F	PAPPA2	TMEM207
FAM3B	PAQR5	TMEM213
FBXO16	PARVG	TMEM45B
FCAMR	PCDHB9	TMEM52B
FGF9	PDK1	TMPRSS2
FLJ22763	PDP2	TNFSF13B
FPR3	PFKFB2	TNFSF8
FREM1	PHYHD1	TRIM59
FTCD	PLA2R1	TRIM71
FUT11	PLEKHA5	TRPM3
FXYD4	PLG	TRPM6
FXYD5	PLPP4	TSPAN33
FYB	PMEPA1	TTC36
GALM	PNPLA3	TTC39B
GAS2L3	PPARA	UPP2
GATM	PPP1R18	USP2
GBP1	PPP1R1A	WDR72
GBP2	PPP1R3B	WLS
GBP5	PPP1R3C	WNK3
GGACT	PRDM1	WNK4
GGT6	PRDM16	XAF1
GGTA1P	PREX1	YIPF5
GIMAP2	PRLR	ZAK
GIT2	PRODH2	ZC3HAV1L
GJC1	PRR11	ZEB2
GLIPR1	PRR15	ZFAS1
GLOD5	PSAT1	ZNF395
GLYAT	PTAFR	ZNF532
		ZNRF3
