# Supplementary figures and images for: G Protein γ subunit 7 loss contributes to progression of clear cell renal cell carcinoma
Source: J Cell Physiol. 2019 Apr 3;234(11):20002–12. doi: 10.1002/jcp.28597 (PMC6767067; doi:10.1002/jcp.28597)

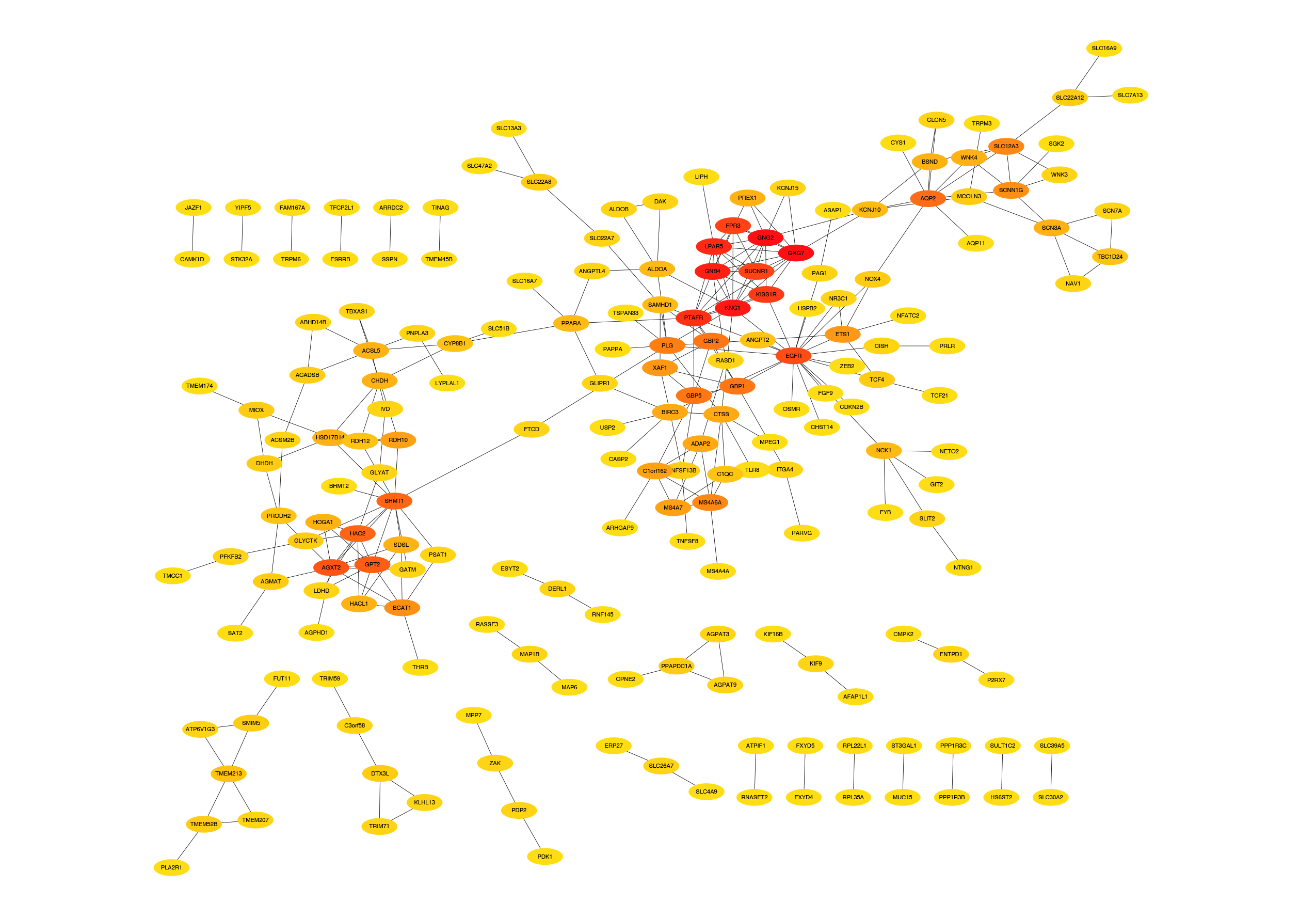

Supplement: Supplementary file 2 — Supporting information [file JCP-234-20002-s002.tif]
