# Supplementary material for: G Protein γ subunit 7 loss contributes to progression of clear cell renal cell carcinoma
Source: J Cell Physiol. 2019 Apr 3;234(11):20002–12. doi: 10.1002/jcp.28597 (PMC6767067; doi:10.1002/jcp.28597)
Supplement: Supplementary file 3 — Supporting information [file JCP-234-20002-s003.pdf]

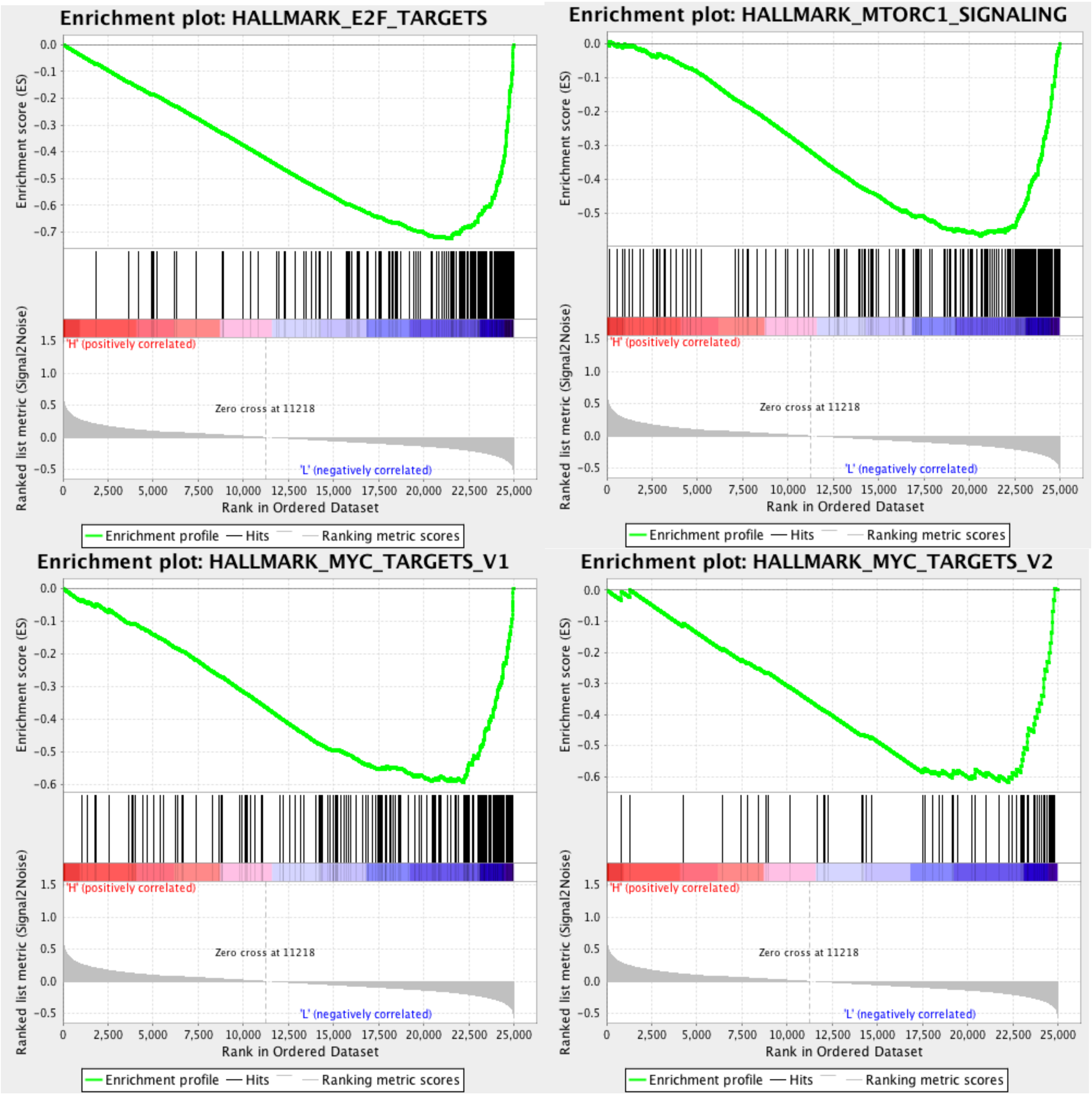
Enrichment plot: HALLMARK_E2F_TARGETS
Enrichment plot: HALLMARK_MTORC1_SIGNALING
Enrichment plot: HALLMARK_MYC_TARGETS_V1
Enrichment plot: HALLMARK_MYC_TARGETS_V2
Enrichment score (ES)
Ranked list metric (Signal2Noise)
'H' (positively correlated)
'L' (negatively correlated)
Zero cross at 11218
Rank in Ordered Dataset
Enrichment profile
Hits
Ranking metric scores

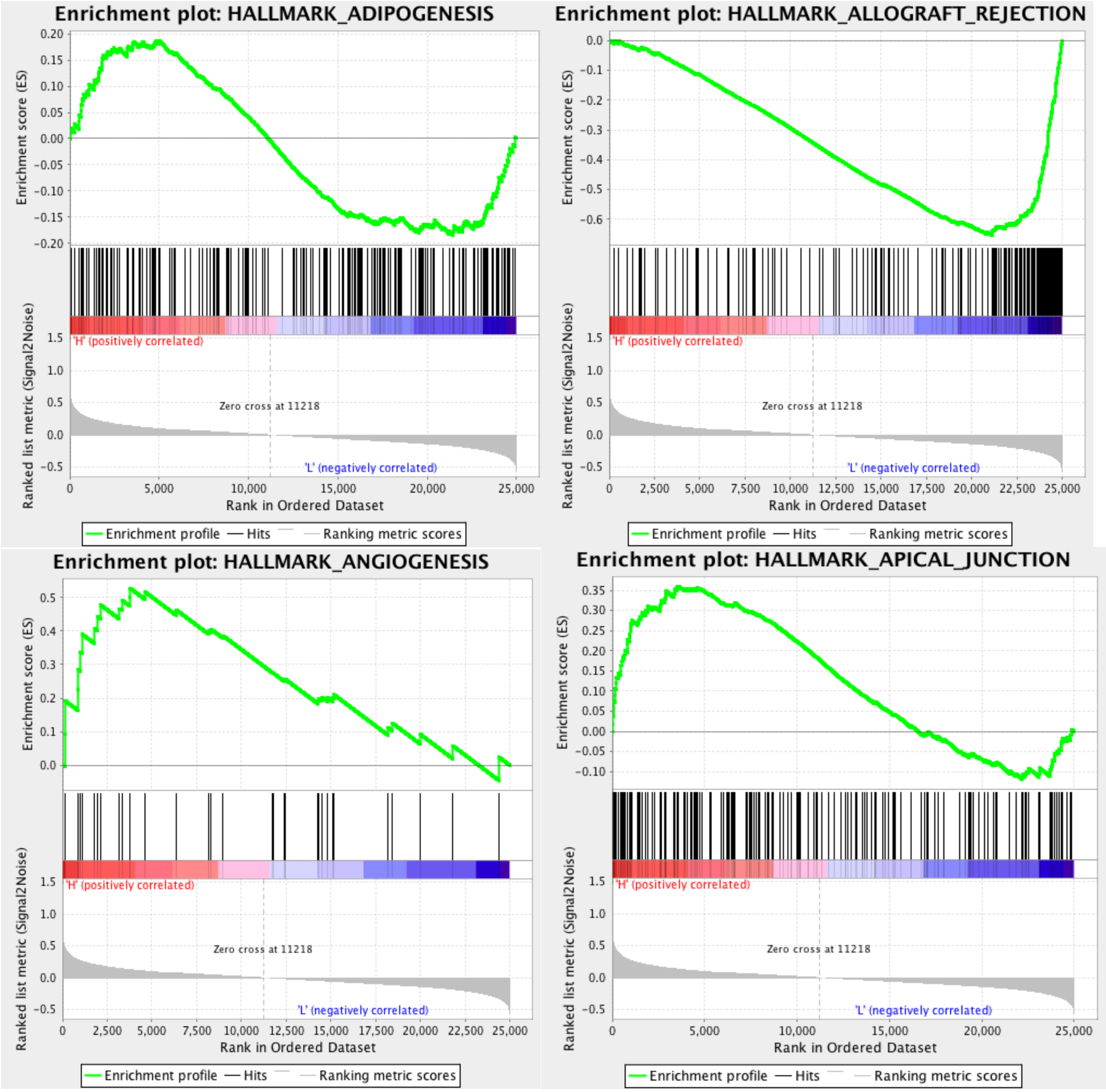
Enrichment plot: HALLMARK_ADIPOGENESIS
Enrichment plot: HALLMARK_ALLOGRAFT_REJECTION
Enrichment plot: HALLMARK_ANGIOGENESIS
Enrichment plot: HALLMARK_APICAL_JUNCTION
Enrichment score (ES)
Ranked list metric (Signal2Noise)
'H' (positively correlated)
Zero cross at 11218
'L' (negatively correlated)
Rank in Ordered Dataset
Enrichment profile
Hits
Ranking metric scores

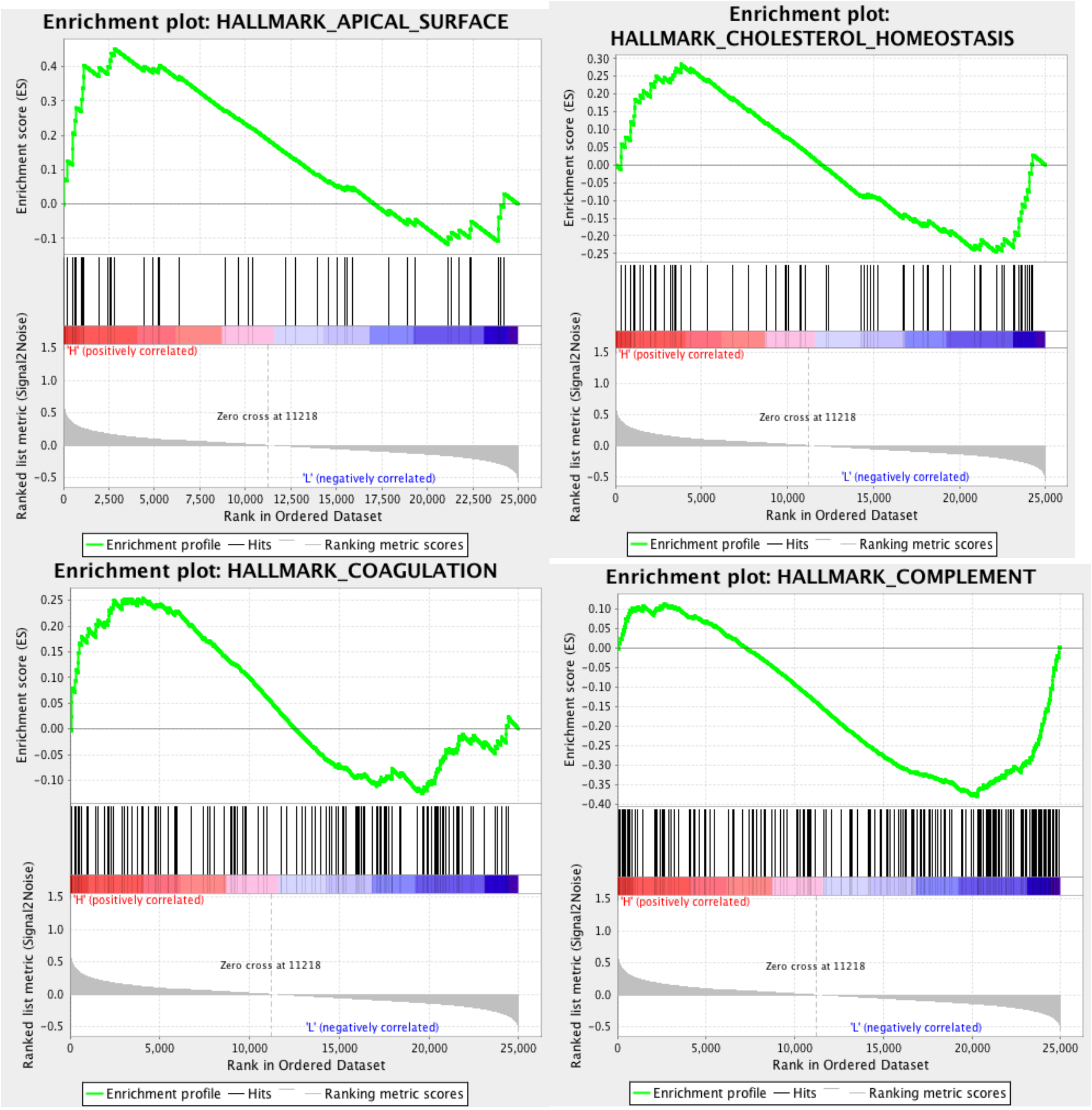
Enrichment plot: HALLMARK_APICAL_SURFACE
Enrichment plot: HALLMARK_CHOLESTEROL_HOMEOSTASIS
Enrichment plot: HALLMARK_COAGULATION
Enrichment plot: HALLMARK_COMPLEMENT
Enrichment score (ES)
Ranked list metric (Signal2Noise)
'H' (positively correlated)
Zero cross at 11218
'L' (negatively correlated)
Rank in Ordered Dataset
Enrichment profile
Hits
Ranking metric scores

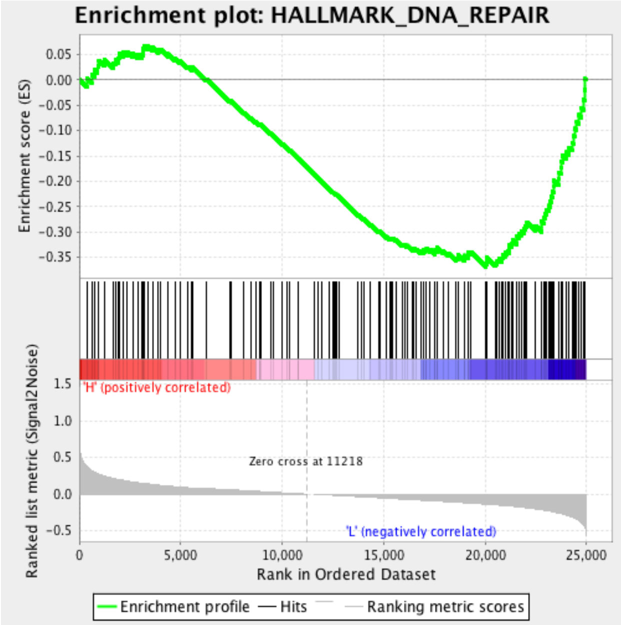
Enrichment plot: HALLMARK_DNA_REPAIR
Enrichment score (ES)
Ranked list metric (Signal2Noise)
'H' (positively correlated)
Zero cross at 11218
'L' (negatively correlated)
Rank in Ordered Dataset
Enrichment profile
Hits
Ranking metric scores

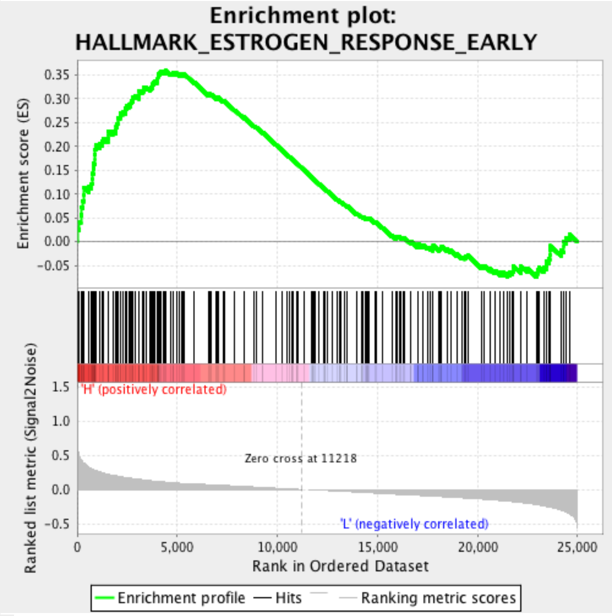
Enrichment plot: HALLMARK_ESTROGEN_RESPONSE_EARLY
Enrichment score (ES)
Ranked list metric (Signal2Noise)
'H' (positively correlated)
Zero cross at 11218
'L' (negatively correlated)
Rank in Ordered Dataset
Enrichment profile
Hits
Ranking metric scores

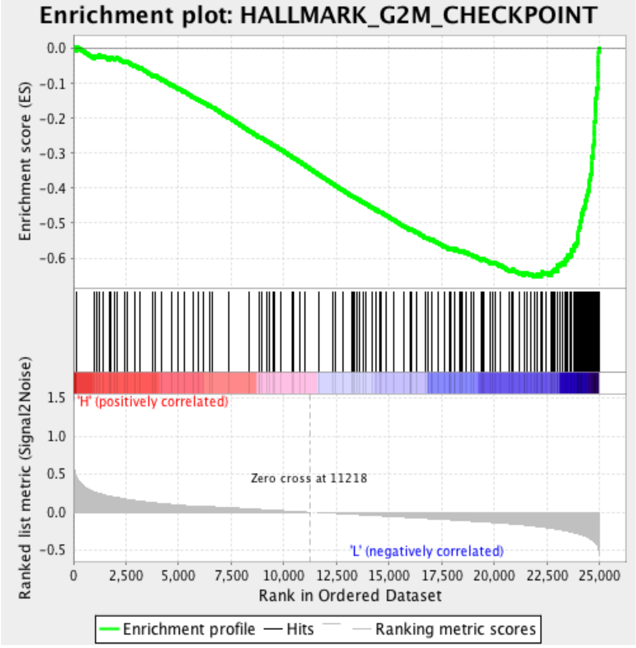
Enrichment plot: HALLMARK_G2M_CHECKPOINT
Enrichment score (ES)
Ranked list metric (Signal2Noise)
'H' (positively correlated)
Zero cross at 11218
'L' (negatively correlated)
Rank in Ordered Dataset
Enrichment profile
Hits
Ranking metric scores

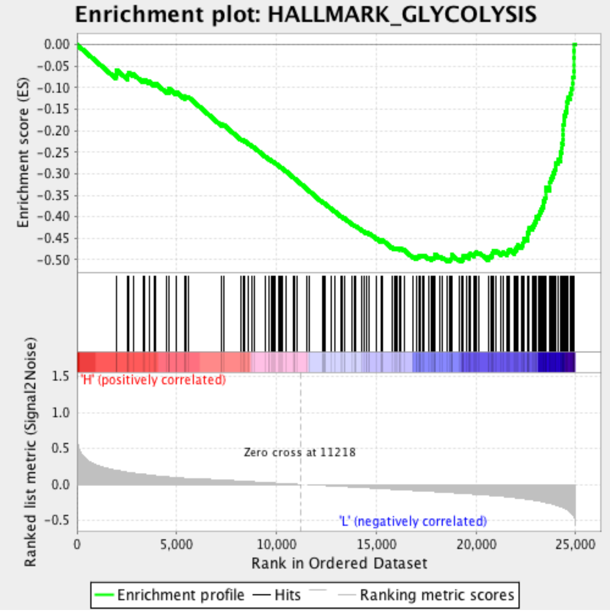
Enrichment plot: HALLMARK_GLYCOLYSIS
Enrichment score (ES)
Ranked list metric (Signal2Noise)
'H' (positively correlated)
Zero cross at 11218
'L' (negatively correlated)
Rank in Ordered Dataset
Enrichment profile
Hits
Ranking metric scores

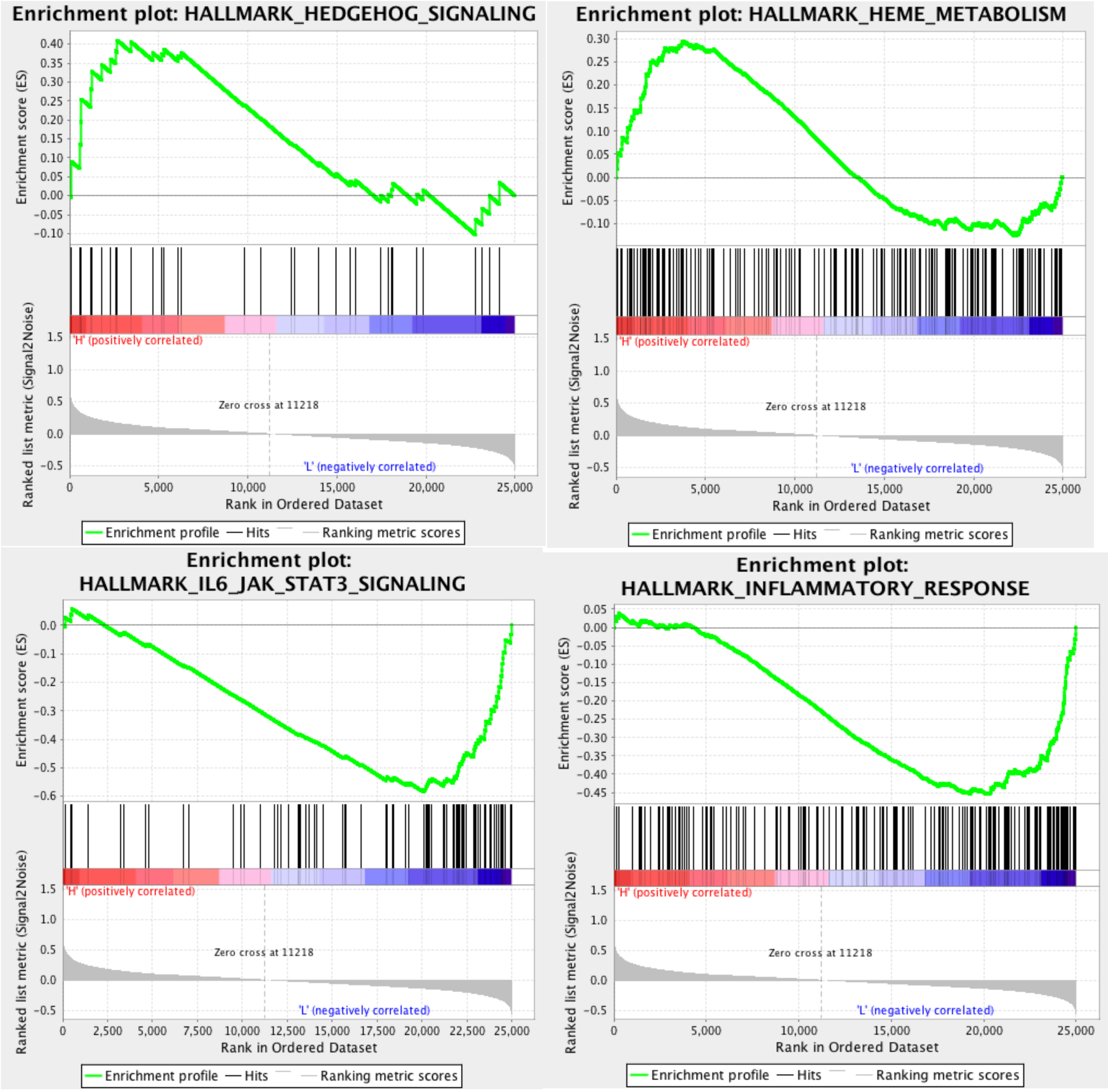
Enrichment plot: HALLMARK_HEDGEHOG_SIGNALING
Enrichment score (ES)
Ranked list metric (Signal2Noise)
'H' (positively correlated)
Zero cross at 11218
'L' (negatively correlated)
Rank in Ordered Dataset
Enrichment profile — Hits — Ranking metric scores
Enrichment plot: HALLMARK_HEME_METABOLISM
Enrichment score (ES)
Ranked list metric (Signal2Noise)
'H' (positively correlated)
Zero cross at 11218
'L' (negatively correlated)
Rank in Ordered Dataset
Enrichment profile — Hits — Ranking metric scores
Enrichment plot:
HALLMARK_IL6_JAK_STAT3_SIGNALING
Enrichment score (ES)
Ranked list metric (Signal2Noise)
'H' (positively correlated)
Zero cross at 11218
'L' (negatively correlated)
Rank in Ordered Dataset
Enrichment profile — Hits — Ranking metric scores
Enrichment plot:
HALLMARK_INFLAMMATORY_RESPONSE
Enrichment score (ES)
Ranked list metric (Signal2Noise)
'H' (positively correlated)
Zero cross at 11218
'L' (negatively correlated)
Rank in Ordered Dataset
Enrichment profile — Hits — Ranking metric scores

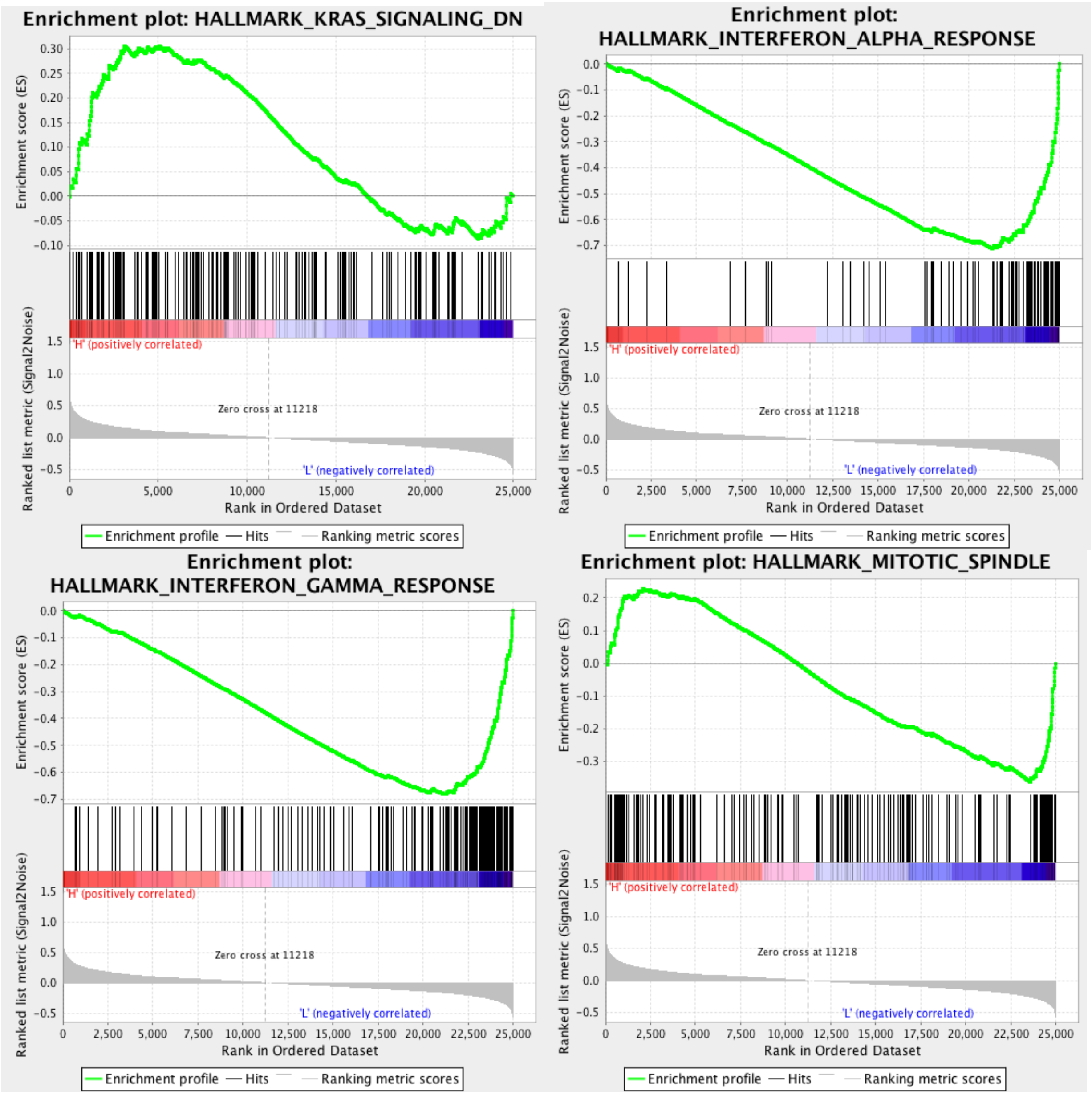

Enrichment plot: HALLMARK_KRAS_SIGNALING_DN
Enrichment plot: HALLMARK_INTERFERON_ALPHA_RESPONSE
Enrichment plot: HALLMARK_INTERFERON_GAMMA_RESPONSE
Enrichment plot: HALLMARK_MITOTIC_SPINDLE
Enrichment score (ES)
Ranked list metric (Signal2Noise)
'H' (positively correlated)
'L' (negatively correlated)
Zero cross at 11218
Rank in Ordered Dataset
Enrichment profile
Hits
Ranking metric scores

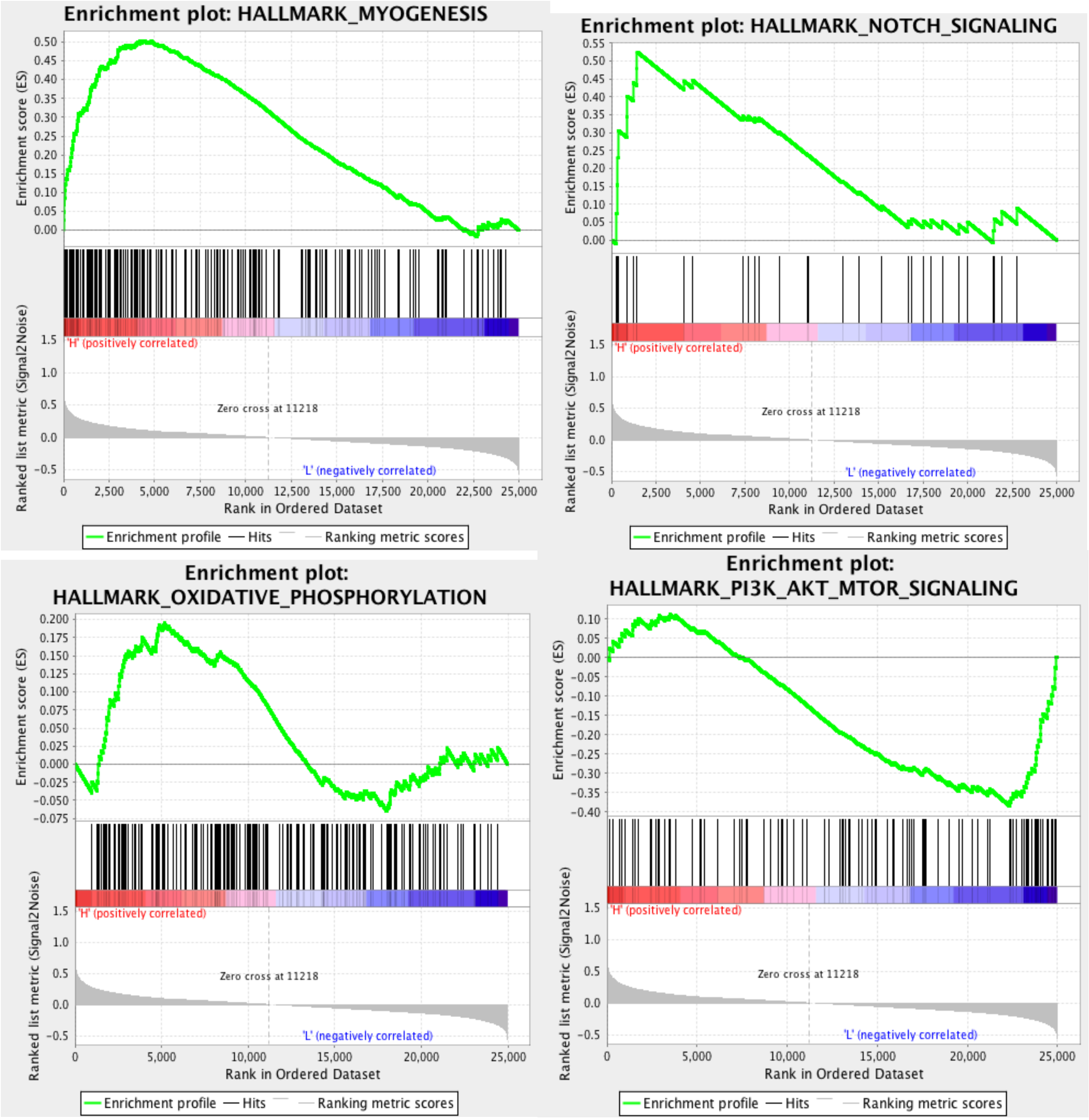
Enrichment plot: HALLMARK_MYOGENESIS
Enrichment score (ES)
'H' (positively correlated)
Zero cross at 11218
'L' (negatively correlated)
Ranked list metric (Signal2Noise)
Rank in Ordered Dataset
Enrichment profile
Hits
Ranking metric scores
Enrichment plot: HALLMARK_NOTCH_SIGNALING
Enrichment score (ES)
'H' (positively correlated)
Zero cross at 11218
'L' (negatively correlated)
Ranked list metric (Signal2Noise)
Rank in Ordered Dataset
Enrichment profile
Hits
Ranking metric scores
Enrichment plot: HALLMARK_OXIDATIVE_PHOSPHORYLATION
Enrichment score (ES)
'H' (positively correlated)
Zero cross at 11218
'L' (negatively correlated)
Ranked list metric (Signal2Noise)
Rank in Ordered Dataset
Enrichment profile
Hits
Ranking metric scores
Enrichment plot: HALLMARK_PI3K_AKT_MTOR_SIGNALING
Enrichment score (ES)
'H' (positively correlated)
Zero cross at 11218
'L' (negatively correlated)
Ranked list metric (Signal2Noise)
Rank in Ordered Dataset
Enrichment profile
Hits
Ranking metric scores

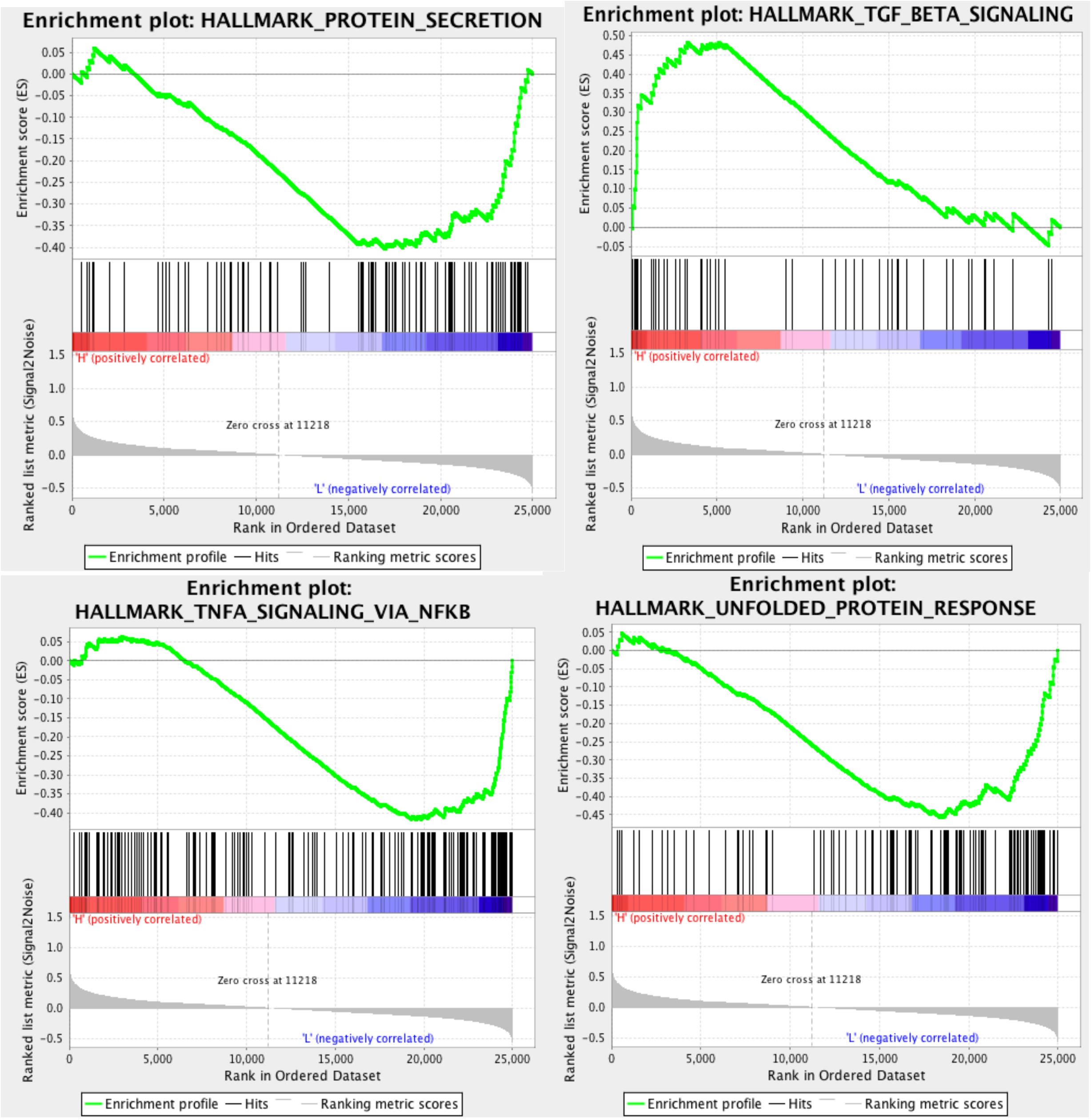
Enrichment plot: HALLMARK_PROTEIN_SECRETION
Enrichment score (ES)
Ranked list metric (Signal2Noise)
'H' (positively correlated)
Zero cross at 11218
'L' (negatively correlated)
Rank in Ordered Dataset
Enrichment profile
Hits
Ranking metric scores
Enrichment plot: HALLMARK_TGF_BETA_SIGNALING
Enrichment score (ES)
Ranked list metric (Signal2Noise)
'H' (positively correlated)
Zero cross at 11218
'L' (negatively correlated)
Rank in Ordered Dataset
Enrichment profile
Hits
Ranking metric scores
Enrichment plot: HALLMARK_TNFA_SIGNALING_VIA_NFKB
Enrichment score (ES)
Ranked list metric (Signal2Noise)
'H' (positively correlated)
Zero cross at 11218
'L' (negatively correlated)
Rank in Ordered Dataset
Enrichment profile
Hits
Ranking metric scores
Enrichment plot: HALLMARK_UNFOLDED_PROTEIN_RESPONSE
Enrichment score (ES)
Ranked list metric (Signal2Noise)
'H' (positively correlated)
Zero cross at 11218
'L' (negatively correlated)
Rank in Ordered Dataset
Enrichment profile
Hits
Ranking metric scores

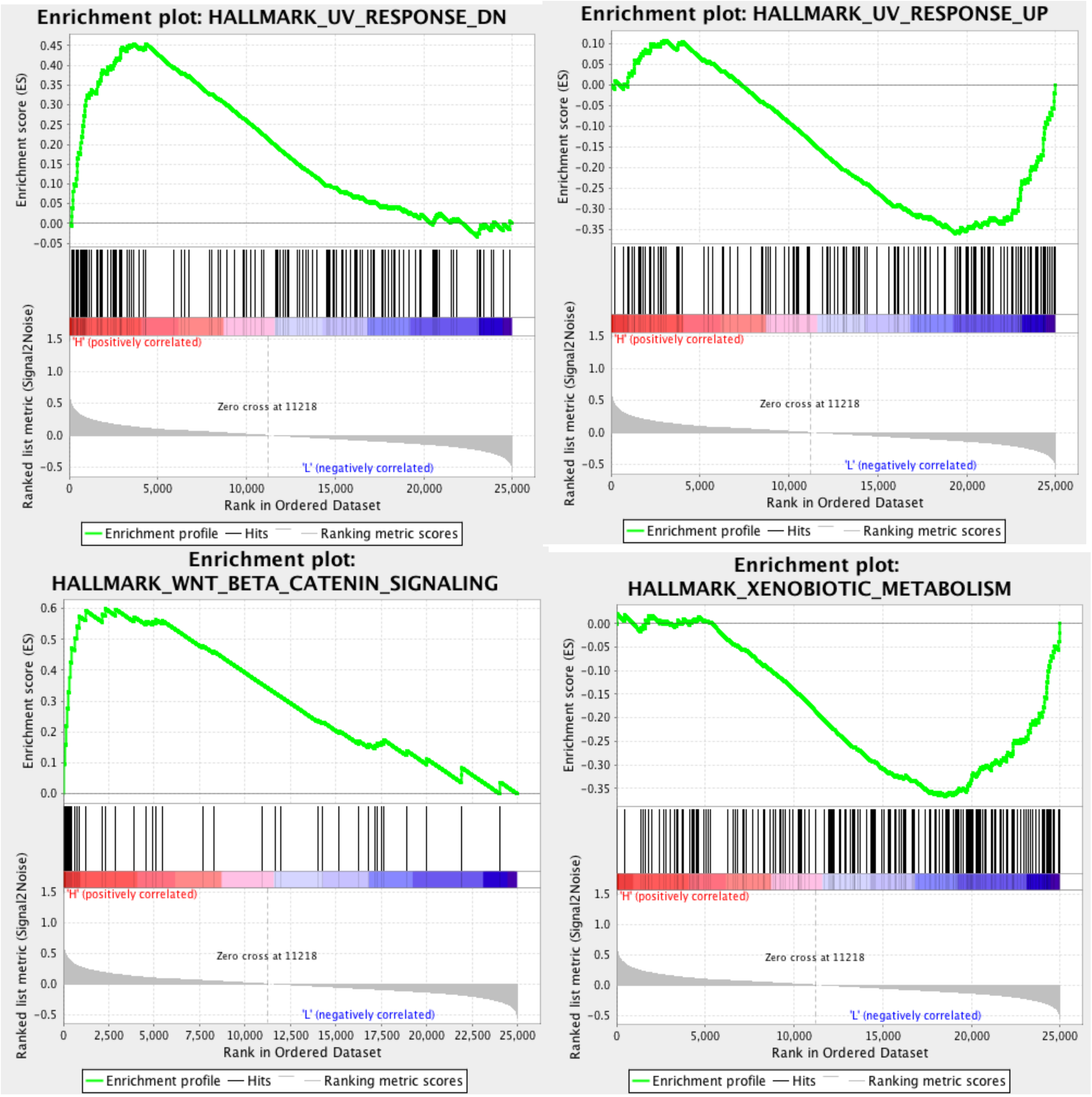
Enrichment plot: HALLMARK_UV_RESPONSE_DN
Enrichment score (ES)
Ranked list metric (Signal2Noise)
'H' (positively correlated)
Zero cross at 11218
'L' (negatively correlated)
Rank in Ordered Dataset
Enrichment profile — Hits — Ranking metric scores
Enrichment plot: HALLMARK_UV_RESPONSE_UP
Enrichment score (ES)
Ranked list metric (Signal2Noise)
'H' (positively correlated)
Zero cross at 11218
'L' (negatively correlated)
Rank in Ordered Dataset
Enrichment profile — Hits — Ranking metric scores
Enrichment plot: HALLMARK_WNT_BETA_CATENIN_SIGNALING
Enrichment score (ES)
Ranked list metric (Signal2Noise)
'H' (positively correlated)
Zero cross at 11218
'L' (negatively correlated)
Rank in Ordered Dataset
Enrichment profile — Hits — Ranking metric scores
Enrichment plot: HALLMARK_XENOBIOTIC_METABOLISM
Enrichment score (ES)
Ranked list metric (Signal2Noise)
'H' (positively correlated)
Zero cross at 11218
'L' (negatively correlated)
Rank in Ordered Dataset
Enrichment profile — Hits — Ranking metric scores
