# Supplementary material for: G Protein γ subunit 7 loss contributes to progression of clear cell renal cell carcinoma
Source: J Cell Physiol. 2019 Apr 3;234(11):20002–12. doi: 10.1002/jcp.28597 (PMC6767067; doi:10.1002/jcp.28597)
Supplement: Supplementary file 4 — Supporting information [file JCP-234-20002-s004.docx]

Supplementary 1: 382 DEGs from GSE781, GSE6344, GSE53757 and GSE66271 cochorts.

Supplementary 2: The PPI network of 382 DEGs was constructed by ctoscape. Significant modules were marked in red, others were marked in yellow.

Supplementary 3: GSEA results of significant changed cell signaling pathway in 50 hallmark gene sets by GNG7 expression.
